# Vitamin status and cognitive function in a long-term care population

**DOI:** 10.1186/1471-2318-5-16

**Published:** 2005-12-13

**Authors:** Lina Paulionis, Sheri-Lynn Kane, Kelly A Meckling

**Affiliations:** 1 Department of Human Health and Nutritional Sciences, University of Guelph, Guelph, Ontario, Canada; 2 St. Joseph's Hospital and Home, Guelph, Ontario, Canada

## Abstract

**Background:**

Ageing can be associated with poor dietary intake, reduced nutrient absorption, and less efficient utilization of nutrients. Loss of memory and related cognitive function are also common among older persons. This study aimed to measure the prevalence of inadequate vitamin status among long-term care patients and determine if an association exists between vitamin status and each of three variables; cognitive function, vitamin supplementation, and medications which alter gastric acid levels.

**Methods:**

Seventy-five patients in a long-term care hospital in Guelph, Ontario were recruited to a cross-sectional study. 47 were female and the mean age was 80.7 (+/-11.5) years, ranging from 48 to 100 years. Blood was used to measure levels of vitamins B12 (cobalamin), B6 (pyridoxal-5'-phosphate/PLP), erythrocyte folate, vitamin B3 (niacin) and homocysteine (Hcy). The Standardized Mini-Mental State Examination (SMMSE) was administered to measure cognitive function. A list of medications and vitamin supplementation for each patient was provided by the pharmacy.

**Results:**

The prevalence of low vitamin (B12, B6, erythrocyte folate, niacin) or high metabolite (homocysteine) levels among 75 patients were as follows: B12 <148 pmol/L in 5/75 (6.7%); B12 between 148 and 221 pmol/L in 26/75 (34.7%); B6 ≤30 nmol/L in 4/75 (5.3%); erythrocyte folate <370 nmol/L in 1/75 (1.3%); niacin ratio ≤1 in 20/75 (26.7%); homocysteine >13.3 μmol/L in 31/75 (41.3%). There was no significant difference among residents grouped into marked (n = 44), mild (n = 14), or normal (n = 9) cognitive function when evaluating the effect of vitamin status. There were no significant differences in mean B12 and homocysteine levels between users and non-users of drug therapy (Losec, Zantac, or Axid). Compared to vitamin supplement non-users, supplemented residents had significantly higher mean B12 (p < 0.0001) and erythrocyte folate (p < 0.05) concentrations and significantly lower mean homocysteine (p < 0.01) levels; 229.1 versus 423.6 pmol/L for B12, 882.9 versus 1043.6 nmol/L for erythrocyte folate and 14.4 versus 12.0 μmol/L for homocysteine.

**Conclusion:**

Given the prevalence data on vitamin status in this sample population, the possible benefits of vitamin supplementation should be considered in clinical intervention studies using these populations of elderly.

## Background

Among independently living, normally aging study populations, evidence exists to support an association between more optimal nutriture (measured by dietary intake or blood parameters) and better performance on cognitive tests [1-3]. Some researchers have suggested that even marginal nutritional status may affect cognitive function [4]. The findings in a six-year follow-up study by La Rue et al [3] showed significant associations between past and concurrent nutrient intakes and better cognitive performance. This would suggest a benefit of a more global diet throughout adulthood.

The role of specific B vitamins in brain related disorders – vitamin B_12 _or niacin in severe cases of cognitive dysfunction [5], folate in depression [6], and vitamin B_6 _in convulsive seizures [7] – has also prompted research on micronutrients and their potential to mitigate cognitive deterioration. Discounting niacin, which has received less attention from researchers, relations of these B vitamins and their metabolic derivative homocysteine (Hcy) to cognitive performance have been demonstrated [8]. Compared to control populations, there also appears to be significantly elevated Hcy [9,10] and low B_12 _and folate levels among Alzheimer disease patients [9].

Levels of vitamin B_12 _[11,12], folate [11] and vitamin B_6 _[13,14] are often insufficient among older persons. For vitamin B_12 _and folate, reduced gastric acid secretion (hypochlorhydria or achlorhydria) from atrophic gastritis [15] or the use of medications [16] impair absorption of these vitamins. For vitamin B_6 _it appears the problem is not an absorptive one, but rather one of cellular uptake or metabolism of the vitamin [17]. Questions have been raised as to whether or not circulating serum vitamin levels are a true measure of deficiency [18]. For this reason, Hcy has been touted as a more reliable measure of deficiency since its metabolism is dependent on functional vitamin B_12_, folate and vitamin B_6 _in tissue. Its usefulness is however limited by genetic, demographic, lifestyle, and pathophysiological factors, all capable of elevating Hcy [19].

Vitamin insufficiencies have been implicated in neurodegenerative disorders and vascular disease; hyperhomocysteinemia already confirmed as an independent risk factor in the latter [20]. With an ever-increasing aging population in North America, ensuring adequate nutriture carries many advantages related to longevity of life and savings in public health resources. To better understand the nutritional needs of institutionalized older persons in Ontario, our study evaluated vitamin status (B_12_, folate, B_6_, niacin, Hcy) and its association with cognitive function, vitamin supplementation and medication use.

## Methods

### Ethics and subject recruitment

Ethics approval was granted by the Research Ethics Board of St. Joseph's Hospital (Hamilton, ON). Information and consent letters outlining the study's objectives and details of subject involvement were sent out to families of residents. St. Joseph's Hospital and Home, located in Guelph Ontario, provides long-term high level care, rehabilitation and out-reach services typical of Type II and III facilities across Canada. Medical and nursing staff gave approval for more cognizant residents to individually consent (verbal or written). Family consent was otherwise required for less independent residents with more severe cognitive impairments. Of the total 127 long-term care residents, 8 residents died during subject recruitment and 44 did not grant consent, because they or their guardians were worried about blood work, guardians thought the resident may be upset by the mini-mental state exam, or were too frail to participate. There were no overall differences between the range of illnesses in participating and non-participating subjects. Seventy-five residents (47 females, 28 males) consented. All 75 participated in the blood work and 67 out of 75 residents partook in the Standardized Mini-Mental State Examination (SMMSE). There were no exclusion criteria for subject involvement given the cross-sectional nature of the study. Of the 75 subjects, 46 had been diagnosed with dementia. Twenty-nine subjects, although not being diagnosed with dementia had several other diagnoses that could affect cognitive function. These included: Parkinson's disease, mental retardation, multiple sclerosis, head injury, transient ischemia attack, hemiplegia, hemiparesis, cerebrovascular attack and seizures. The average age of the subjects was 80.7 yrs (range 48–100) with no difference between males and females. The average duration of long-term care was 1.85 yrs (range 0.1–9) and was not different between males and females.

### Medical and nutritional data

Medical charts were used to collect information on age and gender. A list of medications and vitamin supplementation (oral, intramuscular) was provided by the pharmacy. Medications of interest were proton pump inhibitors (PPIs) (i.e. Losec^®^) and histamine-2 receptor antagonists (H2-blockers) (i.e. Zantac^® ^or Axid^®^). A multivitamin (5000 I.U. vitamin A, 3 mg vitamin B_1_, 2.5 mg vitamin B_2_, 20 mg niacinamide, 1 mg vitamin B_6_, 3 μg vitamin B_12_, 50 mg vitamin C, 400 I.U. vitamin D) and a 1000 μg intramuscular vitamin B_12_injection were considered for vitamin supplementation. Of the 28 subjects using vitamin supplements, 10 had B_12 _injections and 18 took the standard oral multivitamin supplement. Supplement prescriptions were provided by each subject's family doctor and not based on any pre-determined deficiency symptoms. There were no differences between users/non-users of drugs or supplements according to age or gender. Because incidence of specific disease were low for all categories differences in frequency between users and non-users could not be determined.

### Cognitive status

The SMMSE was used to assess the degree of cognitive impairment. To decrease methodological faults and assure methodological reliability, the administrator: 1) Reviewed the SMMSE procedure and grading system outlined in a short booklet and video, 2) Observed a geriatrician conduct the SMMSE on residents not part of the study and 3) Was supervised when conducting the SMMSE on residents not part of the study. The SMMSE was administered on 67 of the 75 residents that gave consent for study participation. Subjects were categorized according to the following: marked cognitive impairment (scores between 0 and 20); mild cognitive impairment (scores between 21 and 25); and normal (scores between 26 and 30). Residents that participated but were mute and/or unresponsive to the administrator's cues received a score of zero out of 30 [21].

### Blood collection and analysis

Blood was collected after an overnight fast at a time when routine annual creatinine measurements would have to be taken. Five tubes of blood were drawn from each resident: One 5 ml SST tube for creatinine and serum B_12_, one 2 ml EDTA tube for erythrocyte folate, two 5 ml EDTA tubes for Hcy and vitamin B_6_, and one 6 ml heparin tube for niacin and methylmalonic acid (analysis incomplete). Blood samples were immediately placed on ice. Blood was collected from six to ten subjects per day over a period of nine days. Within 1.5 hours of blood collection, 100 μL of whole blood from the heparin tube was transferred to a 1.5 ml eppendorf tube; EDTA tubes for Hcy and B_6 _analysis were immediately centrifuged and the plasma transferred into clean tubes. Hcy samples were stored at -20°C, niacin and B_6 _samples at -70°C. Blood samples for creatinine, serum B_12 _and erythrocyte folate analysis were immediately transported to the laboratory for analysis.

Chemiluminescent immunoassays were used for the quantitative determination of vitamin B_12 _and erythrocyte folate (Guelph General Hospital Laboratory Services, Guelph, ON, Canada). It was decided to measure erythrocyte folate rather than serum folate since the former reflects tissue stores of folate and is a more stable marker of folate status. Serum folate is more affected by recent changes in dietary intake [2,21]. The coefficients of variation for cobalamin were as follows: mean value 65 pmol/L (5% CV within run, 8.5% CV for total precision); mean value 276 pmol/L (4.8% CV within run, 6.6% CV for total precision); mean value 572 pmol/L (6.9% CV within run, 7.5% CV for total precision); mean value 719 pmol/L (11.4% CV within run, 11.4% CV for total precision). The coefficients of variation for erythrocyte folate were as follows: mean value 292 nmol/L (2.5% CV within run, 7.8% CV for total precision); mean value 884 nmol/L (2.3% CV within run, 5.9% CV for total precision); mean value 1375 nmol/L (3.1% CV within run, 5.7% CV for total precision); mean value 1948 nmol/L (2.4% CV within run, 5.8% CV for total precision).

Creatinine blood samples were sent to Guelph General Hospital Laboratory, Guelph, ON, Canada (3% CV within run, 4.5% CV for total precision). Creatinine combined with picric acid to produce a colored creatinine-picrate complex. The differential absorbance measured (520 nm and 560 nm) was proportional to creatinine concentration. Creatinine clearance was calculated using the formula of Cockcroft and Gault: [(140-age(years))*weight(kg)]/[serum creatinine (μmol/L)*0.81]. For women, this value was multiplied by 0.85.

Vitamin B_6 _blood samples were sent on dry ice to St. Joseph's Health Centre, Reference Testing Centre, London, ON, Canada (3.5% CV within run, 4.8% CV between runs). Pyridoxal-5'-phosphate (PLP) was detected by HPLC (C_18 _ODS reversed-phase column) and fluorometric detection (325 nm excitation, 400 nm emission) after post column derivatisation with pH 7.5 phosphate buffer containing 1 g/L sodium sulphite. Pyridoxal-5'-phosphate (0 to 200 nmol/L) were used as calibrators. Pyridoxamine 5'-phosphate was used as an internal standard.

Hcy blood samples were sent on dry ice to the Lab Reference Centre, Hamilton General Hospital, Hamilton, ON, Canada. A fluorescence polarization immunoassay was followed for the quantitative measurement of total L-Hcy. Gravimetrically prepared S-adenosyl-L-homocysteine in phosphate buffer (2.5, 5.0, 10.0, 20.0, 50.0 μmol/L Hcy) and phosphate buffer (0 μmol/L Hcy) were used as calibrators. L-Hcy in processed human serum at known concentrations (7.0 μmol/L, 12.5 μmol/L and 25.0 μmol/L) were used as controls. The coefficients of variation were as follows: mean value 5.9 μmol/L (2.2% CV within run, 5.2% CV for total precision); mean value 10.8 μmol/L (1.9% CV within run, 4.1% CV for total precision); mean value 21.6 μmol/L (1.4% CV within run, 3.7% CV for total precision).

Niacin status was measured using the method of Jacobson and colleagues [22]. The coefficients of variation were as follows: NAD (10% CV within and between runs); NADP (11% CV within run and 14% CV between runs).

### Reference values

Low B_12 _levels were defined as <148 pmol/L. Normal levels of B_12_, but at the lower end (low normal), were classified as 148 to 221 pmol/L. Plasma PLP ≤ 30 nmol/L, erythrocyte folate < 370 nmol/L, niacin ratio (NAD/NADP) ≤1, and Hcy >13.3 μmol/L were indicative of low vitamin and elevated metabolite concentrations [22], respectively.

### Statistical analysis

Summary statistics (mean, median, range, 95% confidence interval) was determined using the original (non-transformed) variables. Parametric tests such as analysis of variance, regression models, and independent t-tests were performed on log(base 10) transformed variables to meet the assumption of normal distribution for the continuous variables (vitamin B_12_, vitamin B_6_, erythrocyte folate, niacin number (NAD/NADP*100) and Hcy). In determining the prevalence of vitamin supplementation within SMMSE or drug user groups chi-square analysis was conducted. Probablity values <0.05 were considered statistically significant. All analyses were done by using SPSS computer software (version 7.7).

## Results

Table 1 lists the summary statistics and prevalence data for the vitamins and metabolites. The mean, median and 95% confidence intervals for vitamins B_12_, erythrocyte folate, vitamin B_6_, and niacin number did not fall below their respective reference values of 148 pmol/L, 370 nmol/L, 30 nmol/L, and 100, respectively. The 95% confidence interval of 12.1 to 14.9 μmol/L for Hcy went beyond its cut-off of 13.3 μmol/L. Based on the established reference values, the prevalence of inadequate micronutrient status varied from 1–35% depending on the specific micronutrient examined (Table 1) and almost half of the subjects had elevated Hcy.

**Table 1:** Summary statistics and prevalence data.

	Total (n = 75)
**Vitamin B_12 _(pmol/L)**	
Mean (SD)	301.7 (167.8)
Median	245.0
Range	81.0–982.0
95% CI	263.1–340.3
<148	5 (6.7%)
148–221	26 (34.7%)

**Erythrocyte folate (nmol/L)**	
Mean (SD)	942.9 (367.9)
Median	868.0
Range	316.0–2857.0
95% CI	858.3–1027.5
<370	1 (1.3%)

**Vitamin B_6 _(nmol/L)**	
Mean (SD)	54.3 (21.4)
Median	48.0
Range	28.0–125.0
95% CI	49.4–59.2
≤30	4 (5.3%)

**Hcy (μmol/L)**	
Mean (SD)	13.5 (6.2)
Median	12.5
Range	4.6–48.3
95% CI	12.1–14.9
>13.3	31 (41.3%)

**Niacin Number**	
Mean (SD)	161.3 (81.6)
Median	140.3
Range	33.4–390.5
95% CI	142.5–180.1
Niacin Ratio ≤1	20 (26.7%)

**Figure 1:**
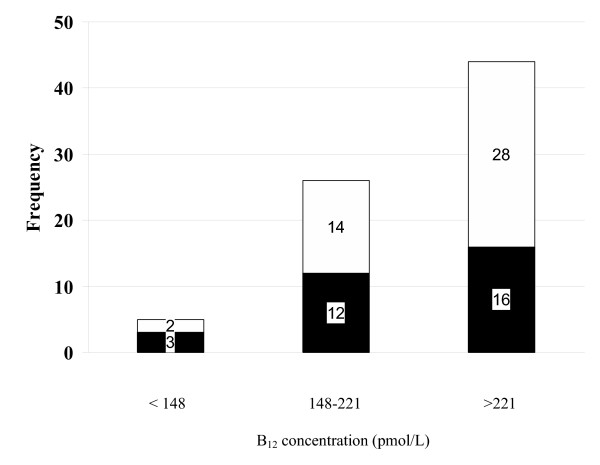
Frequency of subjects with high or normal homocysteine levels as a function of serum B_12 _levels. Darkly shaded area represents the subjects with elevated homocysteine >13.3 μmol/L while lightly shaded area represents subjects with homocysteine ≤13.3 μmol/L for the three vitamin B_12 _intervals.

A multiple linear regression showed vitamin B_12 _to be a significant Hcy predictor (See Table 5). Figure 1 shows the prevalence of Hcy elevations decreasing with increasing cobalamin levels from 60% for B_12 _<148 pmol/L to 46.2% for B_12 _between 148 and 221 pmol/L to 36.4% for B_12 _>221 pmol/L.

**Table 5:** Significance of homocysteine predictors from multiple linear regression.

**Predictor**	**p-value**
Vitamin B_12 _(pmol/L)	p < 0.0001
Erythrocyte folate (nmol/L)	n.s.
Vitamin B_6 _(nmol/L)	n.s.
Age (years)	n.s.
Creatinine (μmol/L)	p < 0.05
Creatinine clearance (ml/min)	n.s.

n.s. – not significantly different

There was no significance when evaluating the main effect of vitamin status on SMMSE categorizations (marked, mild, normal). The prevalence of vitamin supplementation did not significantly differ between the marked, mild and normal SMMSE groups (See Table 2).

**Table 2:** Mean vitamin and metabolite levels according to SMMSE status.

		SMMSE		
	**Marked (0–20) **n = 44	**Mild (21–25) **n = 14	**Normal (26–30) **n = 9	**p-value**

**Age (years)**				
Mean (SD)	84.4 (8.6)*	74.0 (15.1)	75.2 (13.1)	<0.01
**Vitamin B_12 _(pmol/L)**				
Mean (SD)	320.4 (181.1)	284.6 (148.3)	256.9 (99.4)	n.s.
**Erythrocyte folate (nmol/L)**				
Mean (SD)	972.2 (286.5)	730.9 (117.9)	992.7 (471.2)	n.s.
**Vitamin B_6 _(nmol/L)**				
Mean (SD)	54.0 (20.5)	58.1 (28.4)	56.0 (23.6)	n.s.
**Hcy (μmol/L)**				
Mean (SD)	13.9 (6.8)	10.4 (3.0)	14.1 (5.4)	n.s.
**Niacin Number**				
Mean (SD)	166.5 (82.1)	150.2 (58.8)	147.3 (66.1)	n.s.
**Vitamin Supplementation**				
n (%) within each group	17 (38.6%)	5 (35.7%)	4 (44.4%)	n.s.

* p < 0.01 for comparison with mild group

n.s. – not significantly different

There were no significant differences in mean vitamin B_12 _and Hcy levels between users and non-users of drug therapy (PPIs or H2-blockers): 304.6 (128.9) pmol/L and 15.2 (9.2) μmol/L for users, respectively; 300.5 (182.7) pmol/L and 12.8 (4.2) μmol/L for non-users, respectively. Vitamin supplementation between groups did not significantly differ (See Table 3).

**Table 3:** A comparison of vitamin B_12_, Hcy and vitamin supplementation between users and non-users of drug therapy (proton-pump inhibitors and H2-blockers).

	**Drug**	**Therapy**	
	
	**User **(n = 22)	**Non-user **(n = 53)	**p-value**
	
**Vitamin B_12 _(pmol/L)**			
Mean (SD)	304.6 (128.9)	300.5 (182.7)	n.s.
**Hcy (μmol/L)**			
Mean (SD)	15.2 (9.2)	12.8 (4.2)	n.s.
**Vitamin Supplementation**			
n (%) within each group	11 (50.0%)	17 (32.0%)	n.s.

n.s. – not significantly different

Vitamin supplement users had higher mean vitamin B_12 _(p < 0.0001) and erythrocyte folate (p < 0.05) concentrations and lower Hcy (p < 0.01) than non-users (See Table 4). Although the folate status was significantly different, the mean value for both groups was above the reference value of 370 nmol/L.

**Table 4:** Summary statistics and prevalence data for users and non-users of vitamin supplementation.

	**Vitamin**	**Supplementation**	
	**Users **(n = 28)	**Non-users **(n = 47)	**p-value**

**Vitamin B_12 _(pmol/L)**			
Mean (SD)	423.6 (191.6)	229.1 (96.7)	p < 0.0001
≤221	2 (7.1%)	29 (61.7%)	p < 0.0001
**Erythrocyte folate (nmol/L)**			
Mean (SD)	1043.6 (345.8)	882.9 (371.1)	p < 0.05
<370	0	1 (2.1%)	n.s.
**Vitamin B_6 _(nmol/L)**			
Mean (SD)	54.6 (25.3)	54.1 (19.1)	n.s.
≤30	2 (7.1%)	2 (4.3%)	n.s.
**Hcy (μmol/L)**			
Mean (SD)	12.0 (4.9)	14.4 (6.7)	p < 0.01
>13.3	8 (28.6%)	23 (48.9%)	n.s.
**Niacin**			
Mean (SD)	177.7 (79.9)	151.5 (81.8)	n.s.
Niacin Ratio ≤1	4 (14.3%)	16 (34.0%)	n.s.

n.s. not significantly different

The prevalence of inadequate vitamin status was greater for those who were non-users of vitamin supplements compared to users. The group difference was significant for vitamin B_12_; among non-users 29/47 (61.7%) had B_12 _≤221 pmol/L compared to 2/28 (7.1%) among users, p < 0.0001 (See Table 4).

A multiple linear regression model showed creatinine (p < 0.05) and vitamin B_12 _(p < 0.0001) as significant predictors of the criterion homocysteine. Among the other non-significant predictors were creatinine clearance, vitamin B_6_, erythrocyte folate, and age (See Table 5). The amount of variance of the criterion explained by the combined predictors was 41.2%.

## Discussion

The purpose of this study was to examine the frequency of B vitamin deficiencies in a long-term care population in Ontario. Secondary objectives were to examine the relationship, if any, between specific markers of vitamin adequacy and: 1) the use of vitamin supplements, 2) the use of drugs that alter gastric acid secretion, 3) cognitive function as measured by performance on the Standardized Mini-Mental State Examination (SMMSE).

In this study, the prevalence of folate, vitamin B_6 _and niacin deficiency based on established reference values were 1.3%, 5.3% and 26.7%, respectively. Among 177 institutionalized elderly (65–89 years) residing in Mexico, Ortega et al [23] found 6.6% to have erythrocyte folate <360 nmol/L. These authors also found a positive correlation between folate intake and erythrocyte folate (r = 0.28, p < 0.05). The fortification of many foods with folic acid in North America may explain the more optimal folate status in our subjects. In addition to dietary intake differences, geographical differences related to the prevalence of atrophic gastritis could also be considered. Atrophic gastritis creates a low acid environment, which is conducive to bacterial colonization and subsequent bacterial synthesis of absorbable folic acid [25].

The prevalence of vitamin B_6 _≤ 30 nmol/L at 5.3% was low when compared to Joosten et al [26] who reported 51% of 286 hospitalized patients (61–97 years) living in Belgium, Germany or the Netherlands, to have vitamin B_6 _<28.7 nmol/L. Various factors could be considered to explain the difference in prevalence between the two studies including differences in sample size and variable consumption of nutrient rich foods. With respect to the latter, residents of Ontario may be better nourished because of habitual nutrient-rich food intake before and during hospitalization.

Niacin status has not been routinely examined in populations of younger or older adults. Thus there are few reports with which to compare our data. We report here a relatively high prevalence of low niacin status compared to the vitamins B_6 _and folate. The protective effect of niacin nutriture in carcinogenesis [27] prompted researchers towards developing a biomarker for niacin status. With the finding of NAD content in erythrocytes as a sensitive marker for niacin status in humans [28], niacin number (NAD/NADP*100) became a convenient way to evaluate the status of this micronutrient. A mean niacin number of 175 with a 95% confidence interval of 127 to 223 were found among free-living healthy adults and metabolic ward patients [24]. Fu et al [28] suggested a niacin number below 100 (or ratio of NAD/NADP of 1.0) may identify subjects at risk of niacin deficiency. Additionally, analysis of niacin status in different populations revealed 15–20% are niacin deficient [29,30]. Our study found 26.7% at risk of niacin deficiency; a prevalence worthy of greater attention.

Vitamin B_12 _deficiency is estimated to affect 10% to 15% of people over the age of 60 [12]. Serum cobalamin is most commonly used to diagnose deficiency although some have argued it has poor diagnostic efficiency [31]. Pennypacker et al [18] argued that serum vitamin B_12 _was insensitive for screening since similar numbers of geriatric outpatients with low (<148 pmol/L) or low normal (148–221 pmol/L) serum cobalamin had elevated metabolites (>21.3 μmol/L Hcy and >376 nmol/L MMA), 62% and 56% respectively, which fell with cobalamin treatment. Using identical low and low normal cobalamin references ranges, Yao et al [32] reported elevated metabolites (>16 μmol/L Hcy and >270 nmol/L MMA) in 80% and 33% of geriatric outpatients, respectively. For individuals 65 and older with serum cobalamin below 221 pmol/L, the authors recommended analysis of Hcy and MMA, especially in those patients with unexplained hematological and neuropsychiatric disorders [32]. Using serum B_12 _as an estimate of B_12 _vitamin status, we found that fewer than 7% of the hospitalized patients had levels that fell below 148 pmol/L while 34.7% had levels between 148 and 221 pmol/L. Hcy elevations (>13.3 μmol/L) within low (<148 pmol/L) and low normal (148–221 pmol/L) cobalamin ranges were 60% and 46.2%, respectively. Homocysteine and methylmalonic acid (MMA) – both products of reactions requiring cobalamin – carry greater diagnostic utility [31], although the cost of associated assays may hamper their widespread use [31].

Among older people, increased medication use and gastrointestinal changes are factors that affect micronutrient absorption. For vitamins such as cobalamin, mechanisms leading to potential deficiency due to malabsorption include: 1) Inadequate cleavage of protein-bound B_12 _due to hypoacidity (hypochlorhydria/achlorhydria), 2) Decreased secretion of intrinsic factor (a vitamin B_12 _carrier molecule), and 3) Gastric bacterial overgrowth (particularly *Escherichia coli *which absorb vitamin B_12_). The contributing factors to these gastrointestinal changes are pernicious anemia and *Helicobacter pylori *infection, associated with type A and type B atrophic gastritis, respectively [12]. Atrophic gastritis, bacterial overgrowth and medication use (proton pump inhibitors or H2-blockers) are possible explanations. However, the mean B_12 _(304.6 and 300.5 pmol/L) and Hcy (15.2 and 12.8 μmol/L) levels did not significantly vary between users and non-users of drug therapy, respectively. It is thus unlikely these medications would have induced low cobalamin and elevated homocysteine states. Two factors should however be acknowledged in evaluating the potential effect of gastric acid lowering medications on vitamin B_12 _absorption: 1) type of drug (PPI or H2-blocker) and 2) duration of medication use. In a study comparing omeprazole (a PPI) with H2-blocker therapy in Zollinger-Ellison syndrome patients [33], those treated with omeprazole (n = 111; mean duration of 4.5 years) had significantly lower serum B_12 _levels than those treated with H2-blockers (n = 20; mean duration of 10 years). Omeprazole treatment reduced acid secretion to significantly lower levels than H2-blockers. Sustained hypochlorhydria or achlorhydria, primarily occurring in those treated with omeprazole, was the only factor associated with reduced serum B_12 _levels. To explain this difference researchers have noted that PPIs cause prolonged, profound hypochlorhydria [34] whereas the effect of H2 blockers is short lived – a transient hypochlorhydria [35]. Among users of drug therapy (n = 22) in our study population, the majority used H2-blockers (16/22 or 73%) versus PPIs (6/22 or 27%). Duration of medication use was not considered in our study. Studies have shown that prolonged treatment with PPIs (~3.5 years) [36] or H2-blockers [37] is needed to develop low cobalamin levels. The potential for acute medication use and the preferred administration of H2-blockers over PPIs may thus be contributing to the lack of effect of these medications on vitamin B_12 _and Hcy levels. Alternatively, it is possible that the small number of subjects, particularly in the drug-user category, may have obscured differences between the two groups in our study. In future work it would be valuable to have a larger number of drug using subjects for comparison.

Joosten et al [25] estimated 51% of 286 hospitalized elderly to have Hcy elevated >13.9 μmol/L. The only exclusion criteria for this group of subjects were the presence of life-threatening disease. Otherwise, subjects with common geriatric diseases (vascular disease, dementia, diabetes mellitus, osteoporosis, osteoarthritis) were included. Among the factors that significantly correlated with homocysteine were vitamin B_12 _(r = -0.27, p = 0.0001), serum folate (r = -0.38, p = 0.0001), and creatinine clearance (r = -0.44, p < 0.001) [24]. Within our sample population, a 41.3% prevalence of Hcy >13.3 μmol/L was found, the only significant predictors being vitamin B_12 _(p < 0.0001) and creatinine (p < 0.05). If a relationship exists between renal insufficiency and Hcy, it would be more meaningful and reliable to use creatinine clearance over creatinine as a marker of kidney function in older people. This is because body mass declines with age and creatinine clearance is determined irrespective of muscle mass. Creatinine clearance was not a significant predictor of Hcy in our study, implying a lack of effect of renal function on Hcy levels. The significant relationship we found between creatinine and Hcy is in line with what others have observed [38-40].

The dependence of Hcy on vitamin B_12_, vitamin B_6 _and folate as coenzymes in its metabolism explains the significance of B vitamins in cases of elevated Hcy. In fact, Selhub et al found inadequate plasma concentrations of one or more B vitamins (plasma folate, vitamin B_12_, vitamin B_6_) to contribute to 67% of high homocysteine cases (>14 μmol/L) in an elderly population [40]. As mentioned earlier, of the three B vitamins, only vitamin B_12 _was a significant predictor of Hcy in our study. Although vitamin B_6 _has shown weaker associations with Hcy, we expected folate to be a significant predictor [40]. A possibility for folate's non-significance is our selection of erythrocyte folate over serum folate as a status indicator. Koehler et al [41] showed serum Hcy to be negatively correlated with all three folate status indicators (serum, erythrocyte, and dietary intake). However, the authors commented that Hcy corresponded best to short-term folate status as measured by serum folate.

With respect to vitamin B_6_, evidence also exists to suggest a lack of significance between Hcy and vitamin B_6_. Miller et al [42] found no elevation in fasting plasma homocysteine concentrations in studies with animals and humans administered vitamin B_6 _deficient diets. Further, Ubbink et al, showed a non-significant effect of a daily pyridoxine dose on plasma homocysteine concentrations. This was in contrast to folic acid and vitamin B_12 _supplementation, each of which significantly lowered elevated Hcy [43].

Hcy concentrations increase with age [44-46] and the majority of high Hcy cases in an older population can be attributed to vitamin status [40]. A possible explanation for the association between age and Hcy is an age-related decline in enzymes involved in Hcy metabolism [46]. Similar to Koehler et al [41] (100 free living elderly, 68 to 96 years) who found vitamin supplement users to have a significantly better micronutrient profile: 391 vs 292 pmol/L (vitamin B_12_), 1626 vs 1036 nmol/L (erythrocyte folate), and 9.5 vs 11.2 μmol/L (Hcy), we found significantly improved cobalamin (p < 0.0001), erythrocyte folate (p < 0.05), and Hcy (p < 0.01) among supplement users. Additionally, there was a significantly lower prevalence of vitamin B_12 _levels ≤221 pmol/L among supplement users. The composition of the multivitamin in our study included 3 μg of vitamin B_12 _and 1 mg of vitamin B_6_. Some patients were also given an intramuscular cobalamin injection of 1000 μg. Considering the weaker effect of vitamin B_6 _supplementation on Hcy levels [43] and the absence of folic acid in the multivitamin preparation, which has been shown to most effectively reduce Hcy [42,22], it may be that cobalamin was responsible for Hcy lowering in our study, reflecting an intracellular cobalamin deficiency. Rasmussen et al showed a significant average Hcy decrease with cobalamin supplementation alone [46].

Up to 62% of institutionalized older people have dementia [48], and in one study undernutrition was reported in 45.5% of a long-term care population (n = 200) [49]. A multitude of factors – physical, psychological, social and cultural – act on an aged population to reduce dietary intake and alter nutrient metabolism [50]. This demographic is thus at risk of malnutrition, a factor associated with increased morbidity and morality [50]. A study by Litchford et al [51] showed how cognitive dysfunction further exacerbates the already vulnerable nutritional state of an older population. Patients with senile dementia of the Alzheimer's type had lower food intakes and higher energy expenditures compared to cognitively normal elders. A lack of macronutrient repletion in these dementia patients, compounded by a potentially increased micronutrient need (due to alterations in metabolic pathways) could accelerate cognitive decline.

A number of conditions can cause dementia, an aetiology related to nutrition being one of many possibilities. Within our study population there was no association between vitamin status and MMSE category (marked, mild, normal). Whether poor dietary practices lead to cognitive decline or that cognitive decline affects nutritional status, perhaps mediated by metabolic alterations has not been determined. Nevertheless, because poor dietary practices have been shown to influence memory and learning suggests the brain's vulnerability to diet.

To date, there have been no studies of a similar cross-sectional nature conducted on a long-term care population in Ontario. Assessing the prevalence of inadequate vitamin or metabolite status is a starting point for understanding the needs of an aging population and for evaluating the usefulness of therapeutic interventions related to improving vitamin intake. Based on our findings and those of others, we recommend that clinical intervention trials be initiated amongst older populations, both in and out of the hospital setting, to determine the usefulness of this strategy in combating the high frequency of nutrient deficiencies in this population.

## Competing interests

The author(s) declare that they have no competing interests.

## Authors' contributions

Lina Paulionis carried out the recruitment of subjects, applied the MMSE, carried out biochemical analysis and all data analysis and assisted in writing the final manuscript. Sheri-Lynn Kane provided advice as Geriatrician for the study and coordinated all activities at St. Joseph's Hospital and Home, including preparation and presentation of the study for funding and for ethics approval. Dr. Kane also provided expertise in setting cut-off values and final formatting of the manuscript. Kelly Meckling, conceived of the idea for the study, recruited the student and physician to coordinate the study and provided research support and advice throughout the project. Together with Ms. Paulionis they selected the appropriate biochemical and statistical measures to be implemented. Dr. Meckling also worked with the other authors on the final version of the manuscript.

## Pre-publication history

The pre-publication history for this paper can be accessed here:


